# Fabrication and Characterization of Pickering High Internal Phase Emulsions (P-HIPEs) Stabilized by a Complex of Soy Protein Isolate and a Newly Extracted Coix Polysaccharide

**DOI:** 10.3390/foods15010079

**Published:** 2025-12-26

**Authors:** Hong Li, Yubo Cao, Haizhao Song

**Affiliations:** College of Food Science and Engineering, Nanjing University of Finance and Economics/Collaborative Innovation Center for Modern Grain Circulation and Safety, Nanjing 210023, China; 1120220482@stu.nufe.edu.cn (H.L.); 2120191848@stu.nufe.edu.cn (Y.C.)

**Keywords:** pickering high internal phase emulsions, soy protein isolate, coix polysaccharide, stability, rheological behavior

## Abstract

This study explores the fabrication and characterization of Pickering high internal phase emulsions (P-HIPEs) stabilized by soy protein isolate (SPI) and coix polysaccharide (CP) complex. CP exhibited high purity (95.29%) with a molecular weight of 5.53 × 10^5^ Da and was predominantly composed of glucose, as confirmed by monosaccharide analysis and FT-IR spectroscopy. SPI/CP complexes formed well-dispersed nanoparticles with optimal stability at 2% CP concentration, demonstrated by minimal particle size and enhanced zeta potential. P-HIPEs stabilized by these complexes showed excellent physical stability without phase separation or oil leakage, with the creaming index decreasing as particle concentration increased, reaching optimal stability at 12% SPI/CP and pH 9. Particle size and zeta potential measurements indicated smaller, more uniform droplets and intensified electrostatic repulsion under these conditions, effectively preventing droplet coalescence. Confocal microscopy revealed a dense, multilayered interfacial network formed by SPI/CP complexes around oil droplets, enhancing emulsion stability. Rheological analyses confirmed that P-HIPEs exhibited elastic solid-like gel behavior with pronounced shear-thinning and superior thixotropic recovery at 12% SPI/CP and alkaline pH, highlighting improved gel strength and structural integrity. These findings demonstrate the critical influence of SPI/CP concentration and pH on the physicochemical, microstructural, and rheological properties of P-HIPEs, offering valuable insights for developing stable emulsions with enhanced performance and applicability in food systems. Notably, the results emphasize the critical role of SPI/CP concentration and pH in achieving optimal emulsion stability and rheological properties.

## 1. Introduction

Hydrophobic bioactive compounds often face challenges such as poor water solubility, chemical instability, and low bioavailability, which limit their effective delivery and functional performance in food systems [1]. Encapsulation technologies have emerged as a promising strategy to address these challenges. Among these, Pickering emulsions have gained prominence as an innovative delivery platform for hydrophobic bioactives. These emulsions are stabilized by solid particles adsorbed at the oil–water interface, forming a physical barrier that enhances stability and imparts resistance to coalescence, phase separation, and environmental degradation [2]. Pickering high internal phase emulsions (P-HIPEs), a subclass characterized by a high dispersed-phase volume fraction (≥74%), exhibit gel-like rheological properties, high encapsulation efficiency, and controlled release capabilities [3]. These features position P-HIPEs as an ideal delivery system for protecting and delivering bioactives in functional applications [1].

In recent years, natural biopolymers have drawn significant interest as sustainable, biocompatible, and renewable stabilizers for Pickering emulsions [4]. Among them, SPI has been extensively studied for its remarkable emulsifying properties and functional versatility. SPI, a plant-derived globular protein extracted from soybeans, contains a balanced composition of hydrophobic and hydrophilic amino acid residues. This amphiphilic nature enables SPI to effectively adsorb at the oil–water interface, where it reduces interfacial tension and forms a stabilizing layer around dispersed oil droplets [5]. These characteristics make SPI an attractive candidate for stabilizing Pickering emulsions. However, the functionality of SPI as a standalone stabilizer is often compromised under extreme environmental conditions, such as variations in pH, high ionic strength, or elevated temperatures [6,7]. To address the limitations of SPI, researchers have proposed the combination of SPI with polysaccharides.

Coix seed, the seed of the perennial herbaceous plant coix (*Coix lacryma-jobi* L. var. *mayuen Stapf*) has received considerable attention from both the food and medical research sectors, largely due to its numerous health-promoting properties. Research indicates that coix seed is abundant in essential nutrients such as starch, proteins, free amino acids, dietary fibers, vitamins, minerals, phytosterols, and flavonoids, thereby offering significant nutritional value [8]. Coix seed polysaccharides (CP) have garnered significant research interest due to their unique structural characteristics and bioactive properties. These high molecular weight heteropolysaccharides are primarily composed of glucose, galacturonic acid, and mannose, with α-D-glucopyranose (1→6)-linkages, and exhibit bioactive effects such as modulating gut microbiota to activate the IGF1/PI3K/AKT signaling pathway, as well as anti-inflammatory and analgesic activities [9,10,11]. These structural features contribute to CP’s excellent water-holding capacity, film-forming ability, and potential to interact with other biopolymers. CP’s hydrophilic nature and unique structural properties can complement SPI’s amphiphilic characteristics, potentially leading to synergistic effects in emulsion stabilization, improving resistance to environmental stressors such as pH fluctuations, ionic strength changes, and thermal processing. Previous studies have demonstrated that SPI–polysaccharide complexes can form colloidal particles capable of stabilizing Pickering emulsions or HIPEs with improved resistance to creaming and environmental stresses; however, these works have predominantly employed well-known polysaccharides, such as apricot polysaccharide, soybean soluble polysaccharide, and carrageenan [12,13,14]. To the best of our knowledge, there are no reports on the use of SPI/CP complexes for fabricating MCT-based P-HIPEs, nor on systematically elucidating how their concentration and environmental pH jointly modulate emulsion physicochemical, microstructural, and rheological properties. The novelty of the present work lies in introducing structurally characterized CP into SPI-based Pickering systems and providing the first comprehensive evaluation of its functional role in constructing stable, gel-like P-HIPEs for potential food applications.

The physicochemical properties of emulsions, such as droplet size, interfacial composition, and rheological behavior, are critical factors influencing their performance as delivery systems [15,16]. These properties determine the stability of emulsions, the release profile of encapsulated compounds, and their interaction with biological environments. Optimizing these parameters is essential to achieve effective encapsulation and controlled release of bioactives and drugs. Further, the use of medium-chain triglycerides (MCT) as the oil phase in P-HIPEs offers additional advantages. MCTs are widely recognized for their ability to solubilize hydrophobic bioactives, facilitate their absorption, and undergo rapid metabolism in the human body. Incorporating MCTs into the SPI/CP-stabilized P-HIPEs (SPI/CP P-HIPEs) can enhance the emulsions’ structural stability.

In this context, the present study aimed to fabricate P-HIPEs by SPI and CP complexes and systematically investigated the effect of CP concentration and environmental pH on the stability, microstructure, and rheological behavior of the resulting emulsions. The CP was first characterized for molecular weight, monosaccharide composition, and structural features using FT-IR spectroscopy. Subsequently, SPI/CP nanoparticles were prepared and their size, dispersibility, and zeta potential were analyzed to optimize particle properties for P-HIPE stabilization. The physical stability of the emulsions was assessed by visual observation, creaming index, droplet size distribution, and zeta potential measurements across a range of particle concentrations and pH values. Confocal laser scanning microscopy elucidated the interfacial architecture and microstructural features of P-HIPEs, revealing the role of SPI/CP complexes in forming multilayered interfacial films. Finally, comprehensive rheological evaluations provided insights into the gel-like behavior and viscoelastic properties of the emulsions.

This work could contribute to the fundamental understanding of protein–polysaccharide complex-stabilized Pickering HIPEs and highlights the critical parameters governing their stability and functionality. The findings could offer valuable guidance for designing sustainable, food-grade emulsions with tailored textures and improved stability, facilitating their application in advanced food systems, nutraceuticals, and delivery vehicles.

## 2. Materials and Methods

### 2.1. Materials and Chemicals

Coix seeds were obtained from a local market (Nanjing, China). SPI with an 85% protein content was sourced from Beijing Solarbio Science & Technology Co., Ltd. (Beijing, China). MCT, Nile red, and Nile blue, were obtained from Shanghai YuanYe Biotechnology Co., Ltd. (Shanghai, China). All other chemicals were of analytical grade.

### 2.2. Preparation of CP

Coix seeds were pulverized and sieved through a 40-mesh screen. The resulting powder was mixed with anhydrous ethanol at a 1:5 (*w*/*v*) ratio and stirred overnight to precipitate. After discarding the supernatant, the solids were mixed with distilled water at a 1:20 (g/mL) ratio, heated at 90 °C for 2 h, and subjected to sonication for 1 h. The mixture was centrifuged at 8000 rpm for 15 min, and the supernatant was concentrated to one-fourth its original volume under reduced pressure. Four volumes of anhydrous ethanol were added to precipitate the concentrate. The precipitate was redissolved and deproteinized using the Sevage method, involving shaking and repeated centrifugation. The supernatant was dialyzed for 48 h and freeze-dried to yield CP. The total carbohydrate and protein contents were measured using phenol-sulfuric acid [17], and BCA methods, respectively.

### 2.3. Molecular Weight Analysis of CP

The CP sample was prepared at 1 mg/mL and analyzed using HPLC-GFC-ELSD. The analysis was conducted using Agilent 1260 HPLC system (Agilent Technologies, Santa Clara, CA, USA) equipped with a Polysep-GFC-P4000 (Phenomenex Inc., Torrance, California, USA) column (300 × 7.8 mm) at 30 °C, with ultrapure water as the mobile phase at 0.4 mL/min. Each analysis involved an injection volume of 20 μL. Nitrogen was used as the carrier gas at 30 psi, and the ELSD drift tube was set to 100 °C. The molecular weight was determined by comparing the retention time to a calibration curve constructed with dextran standards.

### 2.4. Monosaccharide Composition Analysis of CP

The monosaccharide composition analysis of CP was conducted using the method previously described [18]. Briefly, 5 mg of CP was hydrolyzed with 2 M TFA at 121 °C for 2 h, followed by drying under nitrogen, methanol washing, and reconstitution in sterile water. The hydrolysate and monosaccharide standards were derivatized with PMP by reacting with NaOH and PMP-methanol solution at 70 °C for 1 h. After neutralization and chloroform extraction, the aqueous layer was filtered and analyzed using ThermoU3000 HPLC system equipped with a ZORBAX EclipseXDB-C18 (Agilent Technologies, Santa Clara, CA, USA) column. The mobile phase consisted of potassium phosphate buffer (12 g/L, pH 6.8) and acetonitrile (83:17), with UV detection at 250 nm. Chromatographic conditions included a column temperature of 30 °C, flow rate of 0.8 mL/min, and injection volume of 10 μL. Monosaccharides were identified and quantified by comparing retention times and peak areas with those of standard monosaccharides.

### 2.5. FT-IR Analysis of CP

A total of 1 mg of CP was thoroughly mixed with potassium bromide powder and finely pulverized. The homogeneous mixture was then compressed into 1 mm thick pellets. The FT-IR spectrum was recorded using a Fourier transform infrared spectrophotometer (FT-IR650, Tianjin Gangdong, Tianjin, China) over a spectral range of 4000 to 500 cm^−1^, with a resolution of 4 cm^−1^ and 32 scans. Baseline correction was applied.

### 2.6. Preparation and Characterization of SPI/CP Complex

Protein-anionic polysaccharide conjugates were synthesized following the reported method [19], with some modifications. SPI was dissolved in ultrapure water at a concentration of 4% (*w*/*v*) with stirring at 700 rpm to ensure complete dissolution, followed by hydration at 4 °C for 12 h. The pH was adjusted to 7.0 using 1 M HCl or NaOH, and insoluble materials were removed by centrifugation at 8000 rpm for 25 min, followed by filtration through a 0.22 μm syringe filter. CP solutions at concentrations of 1-6% (*w*/*v*) were prepared in ultrapure water and stirred overnight at room temperature. SPI and CP solutions were combined in a 1:1 (*v*/*v*) ratio by the dropwise addition of CP to SPI under magnetic stirring, and the mixture was heated at 90 °C in a water bath for 1 h to form SPI/CP complex. Additionally, at the optimized CP concentration of 2% (*w*/*v*), the effects of environmental pH (5, 7, and 9) on the formation behavior and colloidal properties of the SPI/CP complexes were further evaluated. The colloidal dispersions were freeze-dried for storage. The particle size, polydispersity index (PDI), and zeta potential of SPI/CP complex were measured using Zetasizer-Nano ZS90 (Malvern Co., Ltd., Worcestershire, UK) at 25 °C with water as the dispersant.

### 2.7. Preparation of P-HIPEs

P-HIPEs were prepared following a previously reported method [20]. For all P-HIPE preparations, freshly prepared SPI/CP dispersions obtained under the optimized conditions (4% SPI, 2% CP, pH 7.0, 90 °C, 1 h) were used directly, without freeze-drying and re-dispersion. MCT was used as the oil phase, with a water-to-oil ratio of 1:3. The pH was adjusted to 7.0, and the mixture was homogenized at 10,000 rpm for 2 min using a high-speed digital homogenizer (FJ-200, Shanghai, China). The resulting P-HIPEs were stored at 4 °C for subsequent experiments.

### 2.8. Optimization of SPI/CP Concentrations and pH in P-HIPEs

The influence of SPI/CP concentrations (4–16% *w*/*v*) on HIPE stability was evaluated at pH 7.0. Furthermore, the stability of SPI/CP was assessed at 12% SPI/CP (*w*/*v*) across pH levels 3, 5, 7, 9, and 11. The creaming index (CI) of P-HIPEs was determined via centrifugation and calculated using the formula: CI (%) = (V2/V1) × 100%, where V1 represented the total P-HIPEs volume and V2 denoted the precipitated water layer volume post-centrifugation.

### 2.9. Characterization of P-HIPEs

The particle size and zeta potential of the P-HIPEs were measured using a Microtrac BLUEWAVE laser particle size analyzer (Microtrac Inc., York, PA, USA) and a Zetasizer-Nano ZS90 (Malvern Co., Ltd., Worcestershire, UK), respectively. The microstructural analysis of the P-HIPEs was carried out using confocal laser scanning microscopy (CLSM) with a Carl Zeiss LSM880 system. Nile Red (0.1%) and Nile Blue (0.1%) were dissolved in carbinol as staining agents; SPI was stained with Nile Blue, and MCT with Nile Red. The P-HIPEs and dyes were mixed in a 25:1 (*v*/*v*) ratio. Nile Red was excited at 488 nm, and Nile Blue at 633 nm.

Rheological properties of the HIPEs gels were assessed using an MCR302 analytical rotational rheometer (Anton Paar GmbH, Graz, Austria), following Bi’s established protocol. Measurements employed parallel plates with a diameter of 50 mm and a gap of 1 mm. The linear viscoelastic region was explored by varying stress from 0.1 Pa to 1000 Pa at 1 Hz, while a frequency sweep from 0.1 Hz to 10 Hz was performed at a constant stress of 1 Pa to maintain linearity. Both the elastic modulus (G′) and the loss modulus (G″) were recorded. Apparent viscosity was determined across a shear rate range of 0.1 s^−1^ to 100 s^−1^. Thixotropic behavior was tested by applying a shear rate sequence of 0.1 s^−1^, 10 s^−1^, and returning to 0.1 s^−1^. Thermal stability was analyzed under a strain of 2.0% and an angular frequency of 10.0 rad/s, with temperatures gradually increased from 30 °C to 80 °C at a rate of 5 °C per minute.

### 2.10. Statistical Analysis

Data were presented as mean ± standard deviation (SD). A one-way ANOVA was applied, and Duncan’s multiple range test was subsequently used to analyze specific differences among the groups. Statistical significance was established at *p* < 0.05 for all analyses.

## 3. Results and Discussion

### 3.1. Molecular Weight and Monosaccharide Composition Analysis of CP

**Figure 1:**
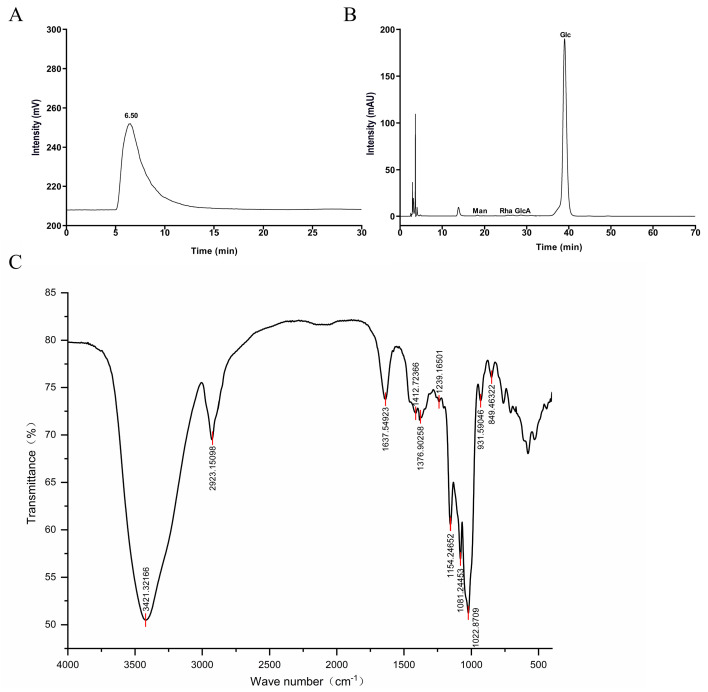
Characterization of CP. Molecular weight (**A**) and monosaccharides composition (**B**) of CP. (**C**) FT-IR spectroscopy of CP.

The compositional analysis of CP revealed a high polysaccharide content (95.29 ± 0.48%) and low protein content (0.62 ± 0.07%), indicating its high purity and potential for functional applications. The molecular weight of CP was determined as 5.53 × 10^5^ Da (Figure 1A), which likely influenced its structural configuration, solubility, protein–carbohydrate interactions, and biological activity [21]. Monosaccharide composition analysis indicated that CP consisted of mannose (Man), rhamnose (Rha), glucuronic acid (GlcA), and glucose (Glc) with a molar ratio of 0.264:0.78:0.167:98.788, respectively, with glucose being predominant (Figure 1B).

### 3.2. FT-IR Spectrometric Analysis of CP

The infrared spectrum of CP, shown in Figure 1C, revealed several key absorption peaks indicative of its structural composition. The peak at 3421.32 cm^−1^ was associated with the O-H stretching vibration, a typical characteristic of carbohydrates [22]. The observed peak at 2923.15 cm^−1^ was attributed to C-H stretching vibrations [23]. The presence of crystalline water was suggested by the absorption peak at 1637.55 cm^−1^. The deformation vibration of the C-H bond was reflected in the peak at 1412.72 cm^−1^ [24], while the absorption at 1376.9 cm^−1^ indicated methyl group (-CH_3_) deformation [25]. The peak at 1239.17 cm^−1^ was assigned to traces of uronic acids and ester sulfate [26]. Absorption peaks at 1081.24 and 1154.25 cm^−1^ suggested the presence of pyranose sugar rings [27]. Additionally, the strong absorbance at 1022.87 cm^−1^ was attributed to stretching vibrations of the pyranose ring in glucosyl residues [28]. The region at 849.46 cm^−1^ indicated the presence of α-configurations [29]. This comprehensive spectral analysis highlighted the complex structural features of CP, which may be relevant to its functional properties and potential applications.

### 3.3. Characteristics of SPI/CP Complex

**Figure 2:**
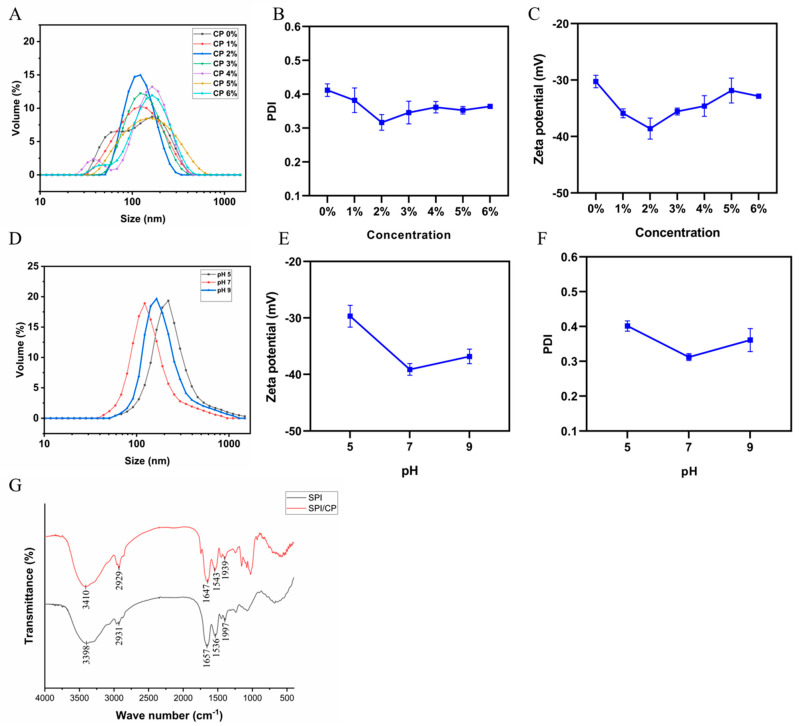
Variations in dynamic particle size distribution (**A**), polydispersity index (PDI) (**B**), and zeta potential (**C**) of SPI/CP particles under different CP concentration levels; Variations in dynamic particle size distribution (**D**), polydispersity index (PDI) (**E**), and zeta potential (**F**) of SPI/CP particles under different CP concentration levels; (**G**) FT-IR spectroscopy of SPI and SPI/CP.

The SPI/CP complex showed unimodal size distributions with polydispersity indices (PDI) below 0.5, indicating good dispersibility (Figure 2A,B). The smallest particle size and lowest PDI were observed at 2% CP, suggesting that CP concentration significantly influenced particle formation. At this concentration, the zeta potential reached its highest absolute value, reflecting enhanced electrostatic interactions that stabilized the particles (Figure 2C). Under the neutral and mildly alkaline conditions used in this study, both SPI and CP were overall negatively charged: SPI carries deprotonated carboxyl groups above its isoelectric point, while CP provides additional negative charges through uronic acid residues. Typically, electrostatic repulsion between similarly charged biopolymers would hinder association. However, in this system, the formation of SPI/CP complexes was driven by thermal treatment (90 °C, 1 h), which facilitated the overcoming of this energy barrier. The heating process induced the unfolding of SPI globular structures, exposing internal hydrophobic groups and reactive amino side chains. This allowed attractive short-range forces-specifically hydrophobic interactions and hydrogen bonding—to dominate over long-range electrostatic repulsion. Furthermore, as evidenced by the FT-IR analysis (Figure 2G), the shifts in amide I and II bands suggest the formation of covalent linkages (e.g., Schiff bases) via the Maillard reaction, locking the structure into stable nanoparticles. Consequently, the SPI/CP complexes exhibited a net negative zeta potential, whose increasing magnitude with higher CP content reflected the greater contribution of anionic polysaccharide to the particle surface. It was important to note that these measurements characterize the properties of the co-assembled nanoparticles formed via heat-induced interaction (90 °C, 1 h), rather than a simple physical mixture of free biopolymers. This was corroborated by two observations: first, the unimodal particle size distribution (PDI < 0.5, Figure 2A) indicated a uniform population of composite particles rather than a heterogeneous mixture of free SPI and CP; second, the FT-IR analysis (Figure 2G) revealed distinct spectral shifts in the amide regions, confirming the formation of specific intermolecular linkages (hydrogen bonding and Maillard-type interactions) between SPI and CP. Thus, the variations in zeta potential and particle size genuinely reflected the surface charge density and hydrodynamic radius of the formed SPI/CP complexes. The more negative zeta potential does not arise from attraction between oppositely charged species, but rather from a strengthened like-charge repulsion between similarly negatively charged particles, which suppresses aggregation and thereby enhances colloidal stability.

However, higher CP concentrations resulted in larger particle sizes, likely due to aggregation or saturation. Under the optimized CP concentration of 2% (*w*/*v*), variation in environmental pH (5, 7, and 9) further affected complex formation, with the smallest particle size, lowest PDI, and highest absolute zeta potential observed at pH 7 (Figure 2D–F), indicating that neutral conditions favored the most compact and electrostatically stable SPI/CP complexes. These findings demonstrated that a CP concentration of 2% produced SPI/CP nanoparticles with optimal stability, emphasizing the importance of protein–polysaccharide interactions in Pickering emulsion design, with neutrality (pH 7) being most favorable. FTIR spectroscopy was employed to elucidate the intermolecular interactions occurring during SPI/CP coacervation (Figure 2G). Compared to native SPI, the complex formed with CP exhibited a distinct red shift in the amide A band, indicating enhanced hydrogen bonding and strengthened intermolecular forces [30]. Furthermore, significant shifts were observed in the characteristic amide I and amide II regions upon CP addition; specifically, the amide I band red-shifted from 1657 to 1647 cm^−1^, while the amide II band blue-shifted from 1536 to 1543 cm^−1^. These spectral alterations are likely attributable to the formation of covalent bonds between carbonyl and amino groups, corresponding to the generation of typical Maillard reaction products such as Schiff base imine and enaminol moieties [31].

### 3.4. Characteristics of SPI/CP P-HIPEs

#### 3.4.1. Visual Appearance and Creaming Index

**Figure 3:**
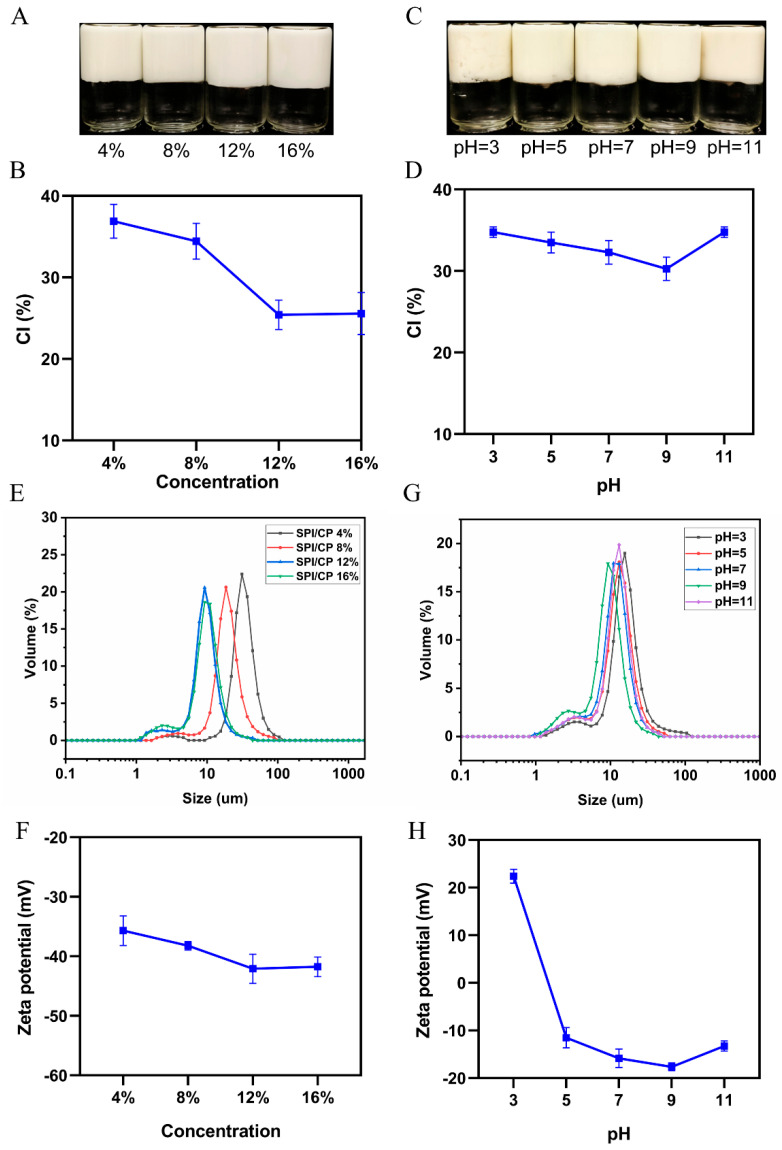
Physical appearance (**A**) and CI (**B**) of SPI/CP P-HIPEs prepared with different SPI/CP concentrations; Physical appearance (**C**) and CI (**D**) of SPI/CP P-HIPEs formulated under varying pH conditions; Dynamic particle size distribution (**E**) and zeta potential (**F**) of SPI/CP P-HIPEs at various SPI/CP concentrations; particle size distribution (**G**) and zeta potential (**H**) of SPI/CP P-HIPEs under different pH conditions. CP, coix seed polysaccharides; SPI, soy protein isolate.

As shown in Figure 3A, P-HIPEs stabilized by SPI/CP particles at concentrations ranging from 4% to 16% exhibited neither emulsification failure nor oil leakage. The CI of the Pickering emulsions decreased with increasing particle concentration (Figure 3B). At lower concentrations, particles were fully adsorbed at the oil–water interface but insufficient in number to entirely encapsulate the droplets, leading to droplet aggregation and larger emulsion droplet sizes. At a concentration of 12%, the increased particle density at the interface formed a thicker interfacial layer, effectively preventing droplet collisions and coalescence, reducing droplet size to its minimum. Further increases in particle concentration did not significantly affect droplet size. Furthermore, the stability of the emulsions improved with increasing pH (Figure 3C). At pH 9, P-HIPEs exhibited the lowest CI, indicating maximal stability under neutral to mildly alkaline conditions (pH 7, 9, and 11) (Figure 3D). However, at pH 3, phase separation occurred, reflecting destabilization in strongly acidic environments. This destabilization is likely due to the reduced solubility and altered charge properties of the SPI/CP particles, which results in weakened adsorption at the oil–water interface. Additionally, the protonation of functional groups in SPI reduces electrostatic repulsion between particles, promoting droplet aggregation and coalescence, ultimately leading to phase separation. These results highlight the pivotal roles of SPI/CP particle concentration and pH in stabilizing HIPEs, with optimal stability achieved at 12% concentration and pH 9.

#### 3.4.2. Particle Size and Zeta Potential

As presented in Figure 3E, increasing SPI/CP concentrations resulted in smaller and more uniform particles. This effect was likely due to enhanced nanoparticle surface wettability during homogenization, facilitating greater adsorption at the oil–water interface, which stabilized the oil droplets. Additionally, SPI/CP increased the viscosity and reinforced the gel network structure of the P-HIPEs, further preventing oil droplet movement and aggregation. The stability of P-HIPEs was also assessed through zeta potential measurements (Figure 3F), which showed higher absolute values with SPI/CP addition, reaching peak at 12% SPI/CP concentration. This increase was attributed to the adsorption of SPI/CP complex nanoparticles at the oil–water interface, enhancing electrostatic interactions and spatial repulsion between them.

The particle size distribution of HIPEs was analyzed at pH values of 3, 5, 7, 9, and 11, revealing that emulsions prepared at pH 9 exhibited the smallest and most uniform particle sizes (Figure 3G), suggesting optimal stability under mildly alkaline conditions. Concurrently, the absolute value of the zeta potential increased with rising pH, peaking at pH 9 (Figure 3H). This trend was attributed to the influence of pH relative to the isoelectric point of SPI nanoparticles. As the pH deviated further from the isoelectric point, the surface charge of the SPI particles intensified, resulting in enhanced electrostatic repulsion among the particles. This repulsion effectively inhibited nanoparticle aggregation and sedimentation, thereby increasing the zeta potential and stabilizing the dispersion. Consequently, higher pH values promoted improved colloidal stability by reinforcing electrostatic interactions and reducing particle aggregation. These results highlight the essential role of pH in modulating particle interactions and dispersibility, ultimately facilitating the stabilization of SPI/CP P-HIPEs with enhanced uniformity and structural integrity.

#### 3.4.3. Microstructure

**Figure 4:**
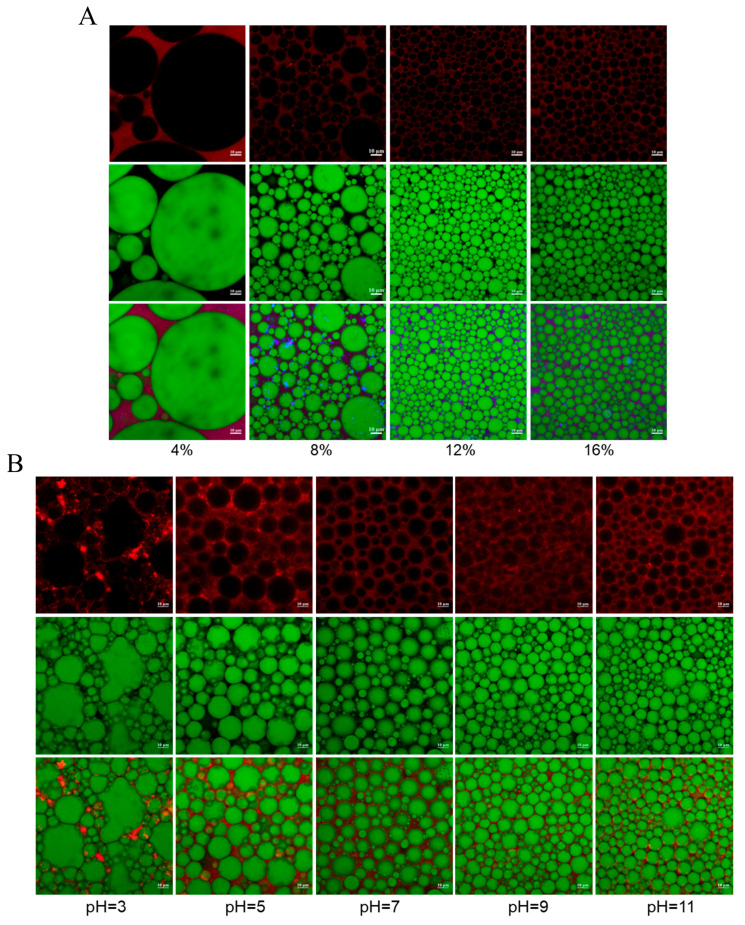
Confocal laser scanning microscopy images of SPI/CP P-HIPEs prepared with various SPI/CP concentrations (**A**) and different pH values (**B**).

The microstructure of P-HIPEs, critical for their stability and digestive properties, was investigated using CLSM. Oil and SPI were selectively stained with 0.1% Nile red and 0.1% Nile blue, respectively, producing green and red signals. As illustrated in Figure 4A, SPI/CP complex formed a robust interfacial layer around spherical oil droplets, and the droplet size decreased as the particle concentration increased. Composite images revealed a multi-layered interfacial structure rich in red and purple signals, indicating a thick, dense network formed by CP-induced SPI aggregation. This multilayered interfacial membrane effectively mitigated droplet coalescence and Ostwald ripening, significantly enhancing emulsion stability. Moreover, at pH 3, diminished red signals at the droplet interface suggested poor SPI adsorption (Figure 4B), likely due to reduced solubility of SPI under acidic conditions [32], thereby weakening interfacial stabilization. At pH levels of 7, 9, and 11, SPI adsorption was markedly enhanced, leading to the formation of smaller and more uniform droplets. This phenomenon can be attributed to the increased solubility and strengthened electrostatic interactions of SPI under neutral and mildly alkaline conditions, which facilitate more robust interfacial adsorption and improved emulsion stability [33]. Overall, the CLSM observations provide direct visual evidence that both SPI/CP concentration and pH govern the thickness and continuity of the interfacial layer and the connectivity of the surrounding gel network, which in turn explain the macroscopic stability trends observed (droplet size, creaming index). These findings underscore that SPI/CP complexes act not only as interfacial particles but also as network-forming agents in the continuous phase, particularly under neutral to mildly alkaline conditions.

#### 3.4.4. Rheological Properties

**Figure 5:**
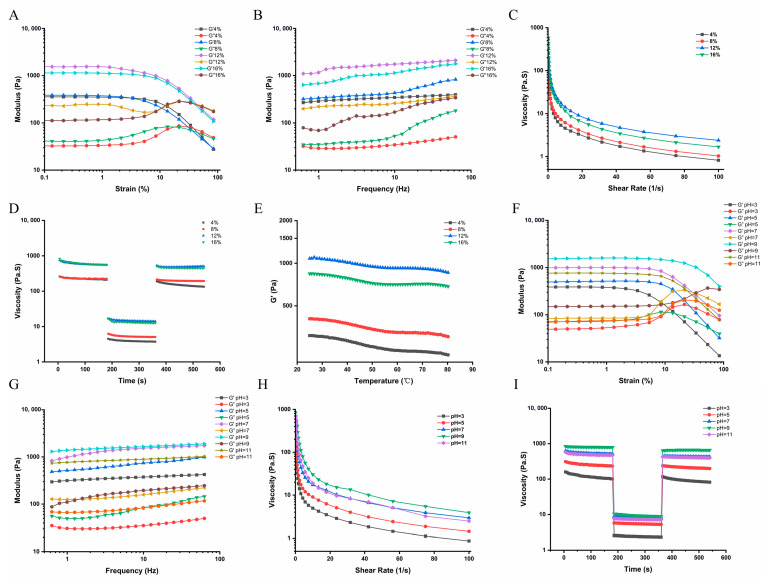
Analysis of the rheological properties of SPI/CP P-HIPEs across different SPI/CP concentration levels: (**A**) Oscillatory stress sweep, (**B**) frequency scan, (**C**) apparent viscosity versus shear rate tests, (**D**) thixotropic recovery, and (**E**) temperature sweep tests. Rheological behavior of SPI/CP P-HIPEs under different pH conditions: (**F**) Oscillatory stress sweep, (**G**) frequency scan, (**H**) apparent viscosity versus shear rate tests, and (**I**) thixotropic recovery tests. G, storage modulus; G″, loss modulus. P-HIPEs, Pickering high internal phase emulsions; CP, coix seed polysaccharides; MCT, medium-chain triglyceride; SPI, soy protein isolate.

The rheological properties of P-HIPEs were investigated to evaluate how SPI/CP concentration and pH affect the internal gel network, which is a critical determinant of the mechanical stability, processability, and textural attributes of HIPE-based food systems. Stress scanning results (Figure 5A) revealed that the storage modulus (G′) of P-HIPEs was significantly higher than the loss modulus (G″) in the linear viscoelastic region, indicating elastic solid gel properties. As SPI/CP concentration increased, the initial G′ linear region widened, and yield stress appeared more gradually, indicating enhanced gel strength. Frequency scans (Figure 5B) demonstrated that G′ and G″ values positively correlated with frequency, with G′ consistently above G″, confirming the elastic-based gel structure of P-HIPEs. This finding aligned with stress scanning and shear rate test results, suggesting that SPI/CP concentration could be manipulated to improve HIPE gel structural properties. Shear rate tests (Figure 5C) showed shear-thinning behavior, characteristic of non-Newtonian fluids [34], with apparent viscosity increasing at higher SPI/CP concentrations due to stronger gel structures. Thixotropic experiments (Figure 5D) revealed a three-stage viscosity response to changing shear rates, with partial structural recovery observed after high shear rates. This recovery was attributed to the directional flow of droplets at high shear rates and their return to disorder at lower rates [35]. Notably, thixotropic recovery performance improved with increasing SPI/CP concentration up to 12%, achieving over 75% recovery, which is considered good for touch effect performance [36]. However, recovery decreased at 16% SPI/CP, suggesting an optimal range for HIPE stability. Temperature increases effects (Figure 5E) further corroborated these results, emphasizing the importance of proportional SPI/CP concentrations for stable HIPE preparation.

The influence of pH on HIPE rheological characteristics was also examined. Stress scanning results (Figure 5F) showed that P-HIPEs prepared at an alkaline pH (9) exhibited higher gel strength, with wider initial G′ linear regions and slower yield stress appearance. Frequency scans (Figure 5G) consistently demonstrated higher G′ and gel strength at pH 9. Viscosity tests (Figure 5H) revealed that apparent viscosity was highest at pH 9, aligning with stress scan results. Moreover, thixotropic recovery (Figure 5I) was strongest at pH 9, indicating optimal HIPE stability under alkaline conditions. Taken together, these rheological results demonstrate that an SPI/CP concentration of 12% at pH 9 produces a strongly elastic, shear-thinning, and partially thixotropic gel network, which is consistent with the observed microstructural features and supports the superior macroscopic stability of the corresponding P-HIPEs.

## 4. Conclusions

This study successfully fabricated Pickering high internal phase emulsions stabilized by soy protein isolate and coix polysaccharide complexes, demonstrating that both particle concentration and pH critically govern emulsion stability, microstructure, and rheological properties. Optimal stability and gel-like behavior were achieved at 12% SPI/CP concentration and pH 9, where enhanced electrostatic repulsion and a robust multilayered interfacial network effectively prevented droplet coalescence. However, limitations include the lack of investigation into long-term storage stability, digestion behavior, and the influence of ionic strength or temperature fluctuations on emulsion performance. Future research should explore these aspects, as well as the potential for tailoring SPI/CP interactions through chemical modifications or incorporation of other bioactive ingredients, to broaden the application scope of such P-HIPEs in functional foods and delivery systems. Moreover, factors such as allergenicity, cost, and environmental impact are crucial considerations when evaluating the feasibility of SPI/CP complexes in food systems, and these aspects should be further investigated in future studies.

## Data Availability

The raw data supporting the conclusions of this article will be made available by the authors on request.

## Abbreviations

BCA, bicinchoninic acid; CI, creaming index; CLSM, confocal laser scanning microscopy; CP, coix polysaccharide; FT-IR, Fourier transform infrared spectroscopy; Glc, glucose; GlcA, glucuronic acid; Man, mannose; MCT, medium-chain triglycerides; PDI, polydispersity index; P-HIPEs, Pickering high internal phase emulsions; PMP, 1-phenyl-3-methyl-5-pyrazolone; Rha, rhamnose; SPI, soy protein isolate; TFA, trifluoroacetic acid.
